# High-Dose IL-2 Skews a Glucocorticoid-Driven IL-17^+^IL-10^+^ Memory CD4^+^ T Cell Response towards a Single IL-10–Producing Phenotype

**DOI:** 10.4049/jimmunol.1800697

**Published:** 2018-12-31

**Authors:** Elizabeth H. Mann, Leona Gabryšová, Paul E. Pfeffer, Anne O’Garra, Catherine M. Hawrylowicz

**Affiliations:** * Medical Research Council and Asthma UK Centre in Allergic Mechanisms of Asthma, King’s College London, London SE1 9RT, United Kingdom;; † Laboratory of Immunoregulation and Infection, The Francis Crick Institute, London NW1 1AT, United Kingdom;; ‡ William Harvey Research Institute, Queen Mary University of London, London EC1M 6BQ, United Kingdom; and; § National Heart and Lung Institute, Faculty of Medicine, Imperial College London, London SW3 6LY, United Kingdom

## Abstract

Glucocorticoids are known to increase production of the anti-inflammatory cytokine IL-10, and this action is associated with their clinical efficacy in asthmatics. However, glucocorticoids also enhance the synthesis of IL-17A by PBMCs, which, in excess, is associated with increased asthma severity and glucocorticoid-refractory disease. In this study, we show that the glucocorticoid dexamethasone significantly increased IL-10 production by human memory CD4^+^ T cells from healthy donors, as assessed by intracellular cytokine staining. In addition, dexamethasone increased production of IL-17A, IL-17F, and IL-22, with the most striking enhancement in cells coproducing Th17-associated cytokines together with IL-10. Of note, an increase in IFN-γ^+^IL-10^+^ cells was also observed despite overall downregulation of IFN-γ production. These dexamethasone-driven IL-10^+^ cells, and predominantly the IL-17^+^IL-10^+^ double-producing cells, were markedly refractory to the inhibitory effect of dexamethasone on proliferation and IL-2Rα expression, which facilitated their preferential IL-2–dependent expansion. Although lower concentrations of exogenous IL-2 promoted IL-10^+^ cells coproducing proinflammatory cytokines, higher IL-2 doses, both alone and in combination with dexamethasone, increased the proportion of single IL-10^+^ T cells. Thus, glucocorticoid-induced IL-10 is only accompanied by an increase of IL-17 in a low IL-2 setting, which is, nevertheless, likely to be protective owing to the induction of regulatory IL-17^+^IL-10^+^–coproducing cells. These findings open new avenues of investigation with respect to the role of IL-2 in glucocorticoid responsiveness that have potential implications for optimizing the benefit/risk ratio of glucocorticoids in the clinic.

## Introduction

Glucocorticoids are a class of lipophilic steroid hormones that are synthesized endogenously by the adrenal cortex. They can bind to the glucocorticoid receptor (GR), which is expressed by most nucleated cells, and trigger a broad range of effects via transactivation and transrepression in addition to other GR-independent actions. Their actions are pleiotropic, affecting various physiological processes including development, metabolism, and inflammation, and, as such, synthetic glucocorticoids have been used in the clinic since 1948 (1). Glucocorticoids remain the most important anti-inflammatory pharmacotherapy in modern medicine despite their untoward side effects. Their anti-inflammatory properties result from their transrepression of proinflammatory genes such as IL-1β and IL-4, transactivation of anti-inflammatory genes, and upregulation of the frequency and activity of regulatory T cells (Tregs) (2). In vivo glucocorticoids have been shown to increase serum levels of the anti-inflammatory cytokine IL-10 (3) as well as the synthesis of IL-10 by cells locally in the airways (4). Furthermore, the synthetic glucocorticoid dexamethasone enhances the concentration of IL-10 in cultures of PBMCs, CD4^+^, and CD8^+^ T cells isolated from healthy humans in vitro (5–8).

The importance of glucocorticoid-induced IL-10 is highlighted by studies in patients with severe steroid-resistant (SR) asthma, who represent a profound clinical challenge for disease management. SR asthma patients have a defect in the dexamethasone-driven IL-10 response (6, 9, 10) and heightened levels of IL-17A; indeed, levels of IL-17A inversely correlate with lung function (11) and are significantly elevated in the peripheral blood (6, 7, 12), sputum (13), serum (14, 15), and bronchial alveolar lining fluid (16, 17) of patients with severe asthma, with the greatest levels observed in patients with SR disease (7). Levels of IL-17A are also elevated in mouse models of airway hyperresponsiveness in which Th17 cells drive pathological conditions (18, 19).

Th17 cells are critical for protecting against mucosal and fungal infections; however, they have also been implicated in various immune-mediated diseases (20). More specifically, cells that differentiate in the presence of IL-23 and TGF-β3 to coexpress Th1- and Th17-associated molecules have been shown to drive experimental autoimmune encephalomyelitis in mice (21, 22). Ramesh et al. (23) showed that human peripheral blood CD4^+^ T cells cultured with IL-23 produced IL-17A, IL-17F, IL-22, and IFN-γ, but not IL-10. However, distinct Th17 phenotypes exist; for example, Zielinski et al. (24) observed *Candida albicans*–specific human CD4^+^ T cells producing IL-17A and IFN-γ, but not IL-10, and *Staphylococcus aureus*–specific cells that, in contrast, could produce IL-10. Functionally, Th17 cells that coproduce IL-10 have been shown to restrain Th17 cell–mediated pathological conditions (21, 25–27).

To better understand these seemingly opposing effects of glucocorticoids on the induction of IL-10 and IL-17A, this study sought to characterize the cellular source of glucocorticoid-driven IL-10 and investigate its coexpression with other cytokines based on emerging data of functionally distinct Th17 subsets. We set out to dissect the mechanism for induction of IL-10 versus IL-17 in T cells, which are critical targets in inflammatory tissues such as the airways in asthma. This study identifies that glucocorticoids enhance the proportion of memory CD4^+^ T cells that coproduce IL-17^+^IL-10^+^, which is reflective of a less inflammatory, protective Th17 phenotype. Notably, addition of higher dose exogenous IL-2 skews this response toward a single IL-10–producing phenotype, which has potential implications for generating more targeted therapeutics.

## Materials and Methods

### Cell isolation and culture

Ethical approval was granted by London Bridge National Research Ethics Service Ethics Committee (REC 09/H0804/77 and REC 14/LO/16990), Human Tissue Act license number 12650 at the Francis Crick Institute, and full written informed consent obtained from all donors. Peripheral venous blood was collected from nonasthmatic male and female healthy adults into syringes containing sodium citrate (10:1). PBMCs were isolated using Lymphoprep as previously described (8). CD45RO^+^ memory CD4^+^ T cells were purified by negative selection (>97% CD4^+^CD45RO^+^; Miltenyi Biotec, Bisley, U.K.). Cells were resuspended at a concentration of 1 × 10^6^ cells/ml in RPMI 1640 growth medium containing 10% FBS, 2 mM l-glutamine, and 50 μg/ml gentamicin.

A total of 0.5 × 10^6^ cells were stimulated with 1 μg/ml plate-bound anti-CD3 (clone OKT-3) and 50 IU/ml recombinant human IL-2 (rhIL-2) (EuroCetus, Harefield, U.K.) for 5 d, unless otherwise stated, in a 48-well plate at 37°C (5% CO_2_). On day 3, half of the supernatant was removed and replaced with full media supplemented with rhIL-2. One hundred nanomolars of dexamethasone (Sigma-Aldrich, Gillingham, U.K.) or 0.01% ethanol vehicle control was added to the cell cultures where indicated. Ten micrograms per milliliter anti–IL-2 (clone 5334; R&D Systems) was added on days 0 and 3 where indicated.

### Cell proliferation

Prior to culture, cells were labeled with 1 μM CellTrace Violet as per the manufacturer’s instructions (Thermo Fisher Scientific). Cell proliferation was assessed by loss of fluorescence intensity on an NxT Attune (Thermo Fisher Scientific) or Fortessa (BD Biosciences).

### Surface staining

Cells were harvested and washed in 2% FCS/PBS prior to performing viability (Zombie Violet/Zombie Aqua viability dye [Life Technologies]) and surface staining on ice in the dark for 30 min. The following Abs were used for cell surface phenotyping: CD4/PerCP/Cy5.5, CD45RA/APCCy7, CD45RO/PECy7 (clones OKT-4, HI100, and UCHL1, respectively; BioLegend), CD3/V500 and CD25/APCH7 (clones SP34-2 and M-A251, respectively; BD Biosciences), and CD122/FITC (TU27; eBiosciences). Cells were washed a further two times in 2% FCS/PBS and then fluorescence was assessed using an NxT Attune or Fortessa.

### Intracellular staining

Human cells were stimulated with 5 ng/ml PMA and 500 ng/ml ionomycin (Sigma-Aldrich) for 4 h at 37°C, with 2 μM monensin added for the final 3 h. Cells were surface stained on ice with the relevant markers and Zombie Aqua viability dye (BioLegend) for 30 min before fixing and permeabilizing using BD Cytofix/Cytoperm Kit as per the manufacturer’s instructions. The following Abs were used to stain intracellular molecules: IL-22/PECy7 (22URTI), IL-22/PE (22URTI), IL-4/PECy7 (8D4-8), IL-17A/APC (eBio64Dec17), IL-17F/aF488 (Poly5166), and IL-2/PerCP/Cy5.5 (MQ1-17H12), all from eBiosciences; IFN-γ/FITC (4S.B3) and IL-10/PE (JEs3-9D7) from BD Biosciences. Cells were washed a further two times in 2% FCS/PBS and then fluorescence was assessed using an NxT Attune or Fortessa.

### Phospho-STAT5 flow cytometry

Cells were rested in full media for 2 h and then stimulated with 5 ng/ml rhIL-2 for 30 min. Samples were then surface stained and fixed prior to resuspending in BD Phosflow Perm Buffer III. Samples were incubated in Perm Buffer III on ice for 30 min and then washed twice in Perm Buffer III before staining with the phospho-STAT5 Ab (Alexa Fluor 488; 47/Stat5 [pY694]; BD Biosciences) for a further 30 min on ice. Cells were washed a further two times in 2% FCS/PBS and then fluorescence was assessed using an NxT Attune or Fortessa.

### Data analysis

Flow cytometry data were analyzed using FlowJo (version 10; Tree Star). Singlet events (based on both forward and side scatter area and height comparison) and viable (Zombie Aqua–negative) cells were selected prior to analysis. Cumulative data analysis was performed in GraphPad Prism version 7.00 for Mac OS X (GraphPad Software, San Diego, CA). After assessing for a Gaussian distribution, an appropriate statistical test was performed as described in the figure legends at the 95% confidence level. Data are shown as mean ± SEM.

## Results

### Dexamethasone increases production of IL-10 by human memory CD4^+^ T cells

**FIGURE 1.**
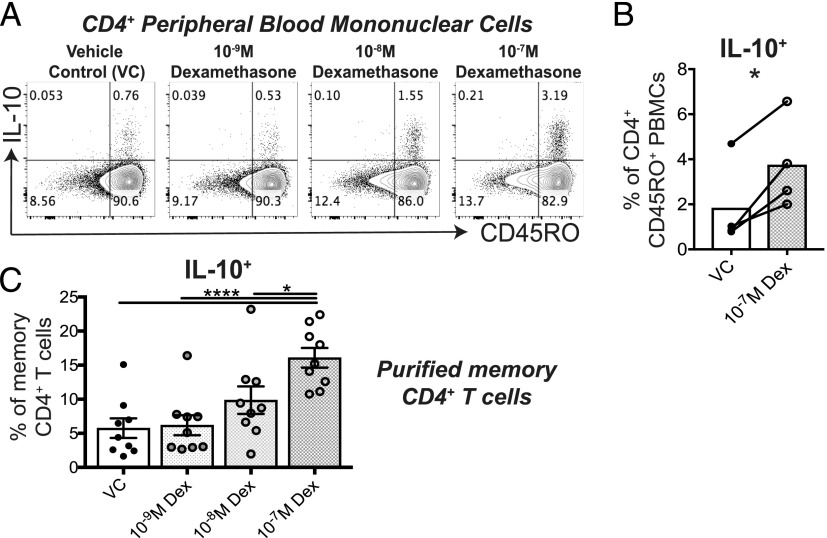
Glucocorticoids enhance production of IL-10 in memory CD4^+^ T cells. PBMCs (**A** and **B**) or memory CD4^+^ T cells (**C**) from healthy donors were stimulated with 1 × 10^−9^ to 1 × 10^−7^M dexamethasone (Dex) or control for 5 d. Cells were then stimulated for 4 h with PMA and ionomycin prior to staining for surface markers and intracellular IL-10. For PBMC cultures, shown are representative plots (A) and cumulative data (B) gating of CD4^+^CD45RO^+^ cells (*n* = 4); data assessed by a paired *t* test. (C) The percentage of IL-10^+^ cells in memory CD4^+^ T cell cultures (*n* = 9); data assessed by repeated measures one-way ANOVA with Tukey multiple comparisons test. *p ≤ 0.05, ****p ≤ 0.0001.

We have previously shown that the glucocorticoid dexamethasone increases the concentration of IL-10 in the supernatants of anti–CD3/IL-2–stimulated, CD8-depleted PBMC, CD4^+^ T cell, and CD8^+^ T cell cultures (5–8). To characterize which T cell subpopulation showed the greatest induction of IL-10 expression by dexamethasone, PBMCs were stimulated with anti-CD3 and IL-2 T cell stimulation in the absence or presence of 10^−7^M dexamethasone. Dexamethasone increased the frequency of IL-10–producing cells to the greatest extent in the CD45RO^+^ memory compartment of CD4^+^ PBMCs following 5 d stimulation, as assessed by intracellular cytokine staining (Fig. 1A, 1B, Supplemental Fig. 1). Consistent with this, in pure memory CD4^+^ T cell cultures, a greater frequency of cells expressed IL-10, and this was dose dependently upregulated by dexamethasone (Fig. 1C).

### Dexamethasone enhances production of IL-10 and IL-17A but not IFN-γ or IL-4

**FIGURE 2.**
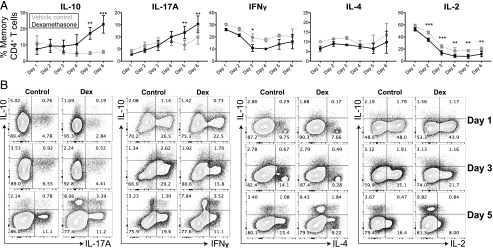
Glucocorticoids increase expression of IL-10 and IL-17A, but not IFN-γ, IL-4, or IL-2, in memory CD4^+^ T cell cultures. Memory CD4^+^ T cells were stimulated in the presence of vehicle control (gray) or 1 × 10^−7^M dexamethasone (black; Dex). On the indicated day, cells were stimulated for 4 h with PMA and ionomycin to assess intracellular cytokine expression. Shown are cumulative data [(**A**); *n* = 4; except IL-4, *n* = 2] and representative plots (**B**). Data assessed by a two-way ANOVA with Sidak multiple comparisons test. *p ≤ 0.05, **p ≤ 0.01, ***p ≤ 0.001.

The kinetics of the dexamethasone-driven IL-10 response was next investigated directly in memory CD4^+^ T cells stimulated over a 6-d culture period (Fig. 2). In the absence of dexamethasone, the frequency of IL-10–producing cells reduced over time. In contrast, addition of 10^−7^M dexamethasone significantly increased the frequency of IL-10^+^ cells by day 5, although not at earlier time points. The proportion of IL-17A^+^ cells gradually increased with time and dexamethasone significantly, albeit more modestly, further enhanced the frequency of IL-17A^+^ T cells on days 5 and 6 of culture (Fig. 2A). In contrast, expression of IFN-γ, IL-4, and IL-2 was reduced or unaltered by dexamethasone throughout the culture (Fig. 2A, 2B). These findings are in keeping with our previous findings (6, 7, 12) and further demonstrate that memory CD4^+^ T cells are the cellular source of both IL-10 and IL-17A following dexamethasone treatment.

### Dexamethasone induces coproduction of IL-10 with multiple proinflammatory cytokines

**FIGURE 3.**
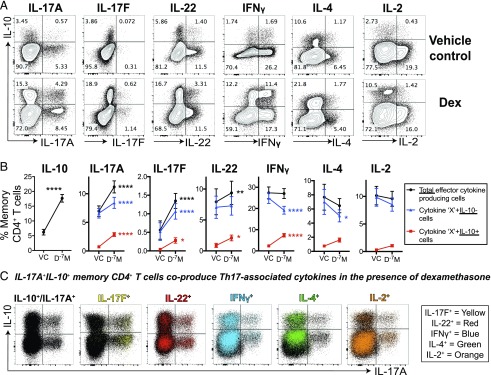
Coproduction of IL-10 and proinflammatory cytokines is induced by glucocorticoids in memory CD4^+^ T cells. Memory CD4^+^ T cells were stimulated in the presence of vehicle control or 1 × 10^−7^M dexamethasone (Dex; D^−7^M). On day 5, cells were stimulated for 4 h with PMA and ionomycin prior to performing intracellular cytokine staining. (**A**) Representative contour plots. (**B**) The total (black) percentage of cells producing the indicated cytokine alongside those coexpressing IL-10^+^ (red) or the IL-10^−^ counterparts (blue); data assessed by two-way ANOVA with Sidak multiple comparisons test (IL-10, *n* = 32; IL-17A, *n* = 29; IL-17F, *n* = 7; IL-22, *n* = 6; IFN-γ, *n* = 25; IL-4, *n* = 6; IL-2. *n* = 19). (**C**) Representative overlay dot plots identifying which cells within the IL-10/IL-17A plots (black; far left) coproduced the indicated cytokine in color (IL-17F in yellow, IL-22 in red, IFN-γ in blue, IL-4 in green, and IL-2 in orange). *p ≤ 0.05, **p ≤ 0.01, ****p ≤ 0.0001.

IL-10^+^CD4^+^ T cells can coproduce multiple other proinflammatory cytokines in a heterogeneous manner (28), including IL-17A (21, 24–27). We, therefore, investigated the effect of dexamethasone on other Th17-associated cytokines, as well as the coproduction of cytokines with IL-10 by memory CD4^+^ T cells. Dexamethasone enriched the proportion of memory CD4^+^ T cells producing the Th17 family members IL-17F and IL-22 (Fig. 3A, 3B, Supplemental Fig. 2A). The average fold change in IL-17A, IL-17F, and IL-22 expression was 1.9-, 4.3-, and 1.4-fold respectively, and that of IL-10^+^ cells was 4.9-fold, relative to the vehicle control (Supplemental Fig. 2B). Notably greater fold increases were consistently observed in cells coproducing IL-10 together with Th17-associated cytokines, as compared with their IL-10^−^ counterparts (Supplemental Fig. 2B). Dexamethasone significantly reduced the frequency of IFN-γ^+^ cells that did not coproduce IL-10 but induced a 7.5-fold increase in the proportion of IFN-γ^+^IL-10^+^ memory CD4^+^ T cells, resulting in no change in the overall proportion of IFN-γ^+^ memory CD4^+^ T cells. A similar trend was observed for production of IL-4 and IL-2 by dexamethasone-treated memory CD4^+^ T cells, with an enhanced proportion of cells coproducing IL-10 in contrast to a reduced frequency of IL-10^−^ cells.

Because it has been reported that inflammatory Th17 cells may coproduce inflammatory cytokines such as IFN-γ (21–23), the dexamethasone-stimulated cells analyzed for IL-10 and IL-17A production were further investigated for coproduction of IL-17F (yellow), IL-22 (red), IFN-γ (blue), IL-4 (green), and IL-2 (orange) (Fig. 3C). As predicted, IL-17F (yellow) was predominantly produced by IL-17A^+^ cells, irrespective of whether they were IL-10^−^ or IL-10^+^. Although IL-22^+^ cells (red) were present in both the IL-10^+^ and IL-10^−^ populations, most did not coproduce IL-17A. The majority of IFN-γ^+^ (blue) or IL-4^+^ (green) cells did not coproduce IL-17A but many coexpressed IL-10. IL-2^+^ (orange) cells were predominantly negative for IL-17A and IL-10 (Fig. 3C). Therefore, dexamethasone dominantly drives the production of IL-10 across all memory CD4^+^ T cell subsets, excluding those producing IL-2. Notably, the dexamethasone-stimulated, IL-17A–producing cells were predominantly negative for IFN-γ, IL-4, and IL-2, although a large proportion coproduced IL-10, which is indicative of a nonpathogenic Th17 phenotype (21, 25–27).

### IL-10^+^ cells are refractory to the suppressive effects of dexamethasone on cell proliferation

**FIGURE 4.**
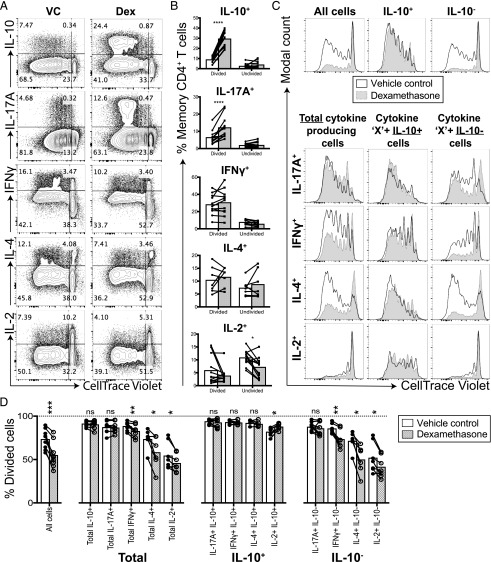
IL-17A^+^ cells and cells coproducing IL-10 are refractory to the suppressive effects of glucocorticoids on cell proliferation. Memory CD4^+^ T cells were labeled with CellTrace Violet on day 0 and then stimulated in the presence of vehicle control (white) or in the presence of 1 × 10^−7^M dexamethasone (Dex; gray). On day 5, cells were stimulated for 4 h with PMA and ionomycin prior to performing intracellular cytokine staining. (**A**) Representative plots showing expression of cytokines relative to cell proliferation. (**B**) Cumulative data of the percentage of cells expressing the indicated cytokine within the divided or undivided cell population; data assessed by a two-way ANOVA with Sidak multiple comparisons test. (**C**) Representative histograms showing the proliferation of cells when gating on the indicated cytokine-expressing populations, alongside cumulative data. (**D**) Data assessed by a paired *t* test. IL-17A and IL-10, *n* = 9; IFN-γ and IL-2, *n* = 7; IL-4, *n* = 6. *p ≤ 0.05, **p ≤ 0.01, ***p ≤ 0.001, ****p ≤ 0.0001.

To investigate the mechanisms driving the enrichment of IL-10^+^ cells within dexamethasone-treated memory CD4^+^ T cell cultures, cytokine expression was assessed in relation to cell proliferation. Memory CD4^+^ T cells were labeled with the fluorescent dye CellTrace Violet prior to stimulation in the presence or absence of dexamethasone. IL-10 and IL-17A were produced predominantly by cells that had undergone multiple rounds of cell division (Fig. 4A) and were enhanced in the presence of dexamethasone (Fig. 4A, 4B). In contrast, IFN-γ and IL-4 production was seen in both dividing and nondividing cells, but their frequency was not altered by dexamethasone. IL-2 was produced primarily by the undivided population and was decreased by dexamethasone (Fig. 4A, 4B).

Glucocorticoids have been repeatedly shown to dampen T cell proliferation (29), which we now show for total memory CD4^+^ T cells (“all cells,” Fig. 4C, 4D). However, cells producing IL-10 and/or IL-17A were refractory to this suppressive effect of dexamethasone on cell proliferation (Fig. 4C, 4D). This contrasts with the proliferation of IFN-γ^+^, IL-4^+^, and IL-2^+^ cells, which was significantly reduced in dexamethasone-treated cultures (Fig. 4C, 4D). Notably, the proliferation of all IL-10^+^ memory CD4^+^ T cells, regardless of whether they coproduced IFN-γ, IL-4, or IL-2, was not suppressed by dexamethasone, in marked contrast to their IL-10^−^ counterparts (Fig. 4C, 4D). These data show that IL-17A^+^ and IL-10^+^ cells are markedly refractory to the antiproliferative effects of dexamethasone.

### IL-10^+^ memory CD4^+^ T cells are resistant to downregulation of the IL-2R by dexamethasone

**FIGURE 5.**
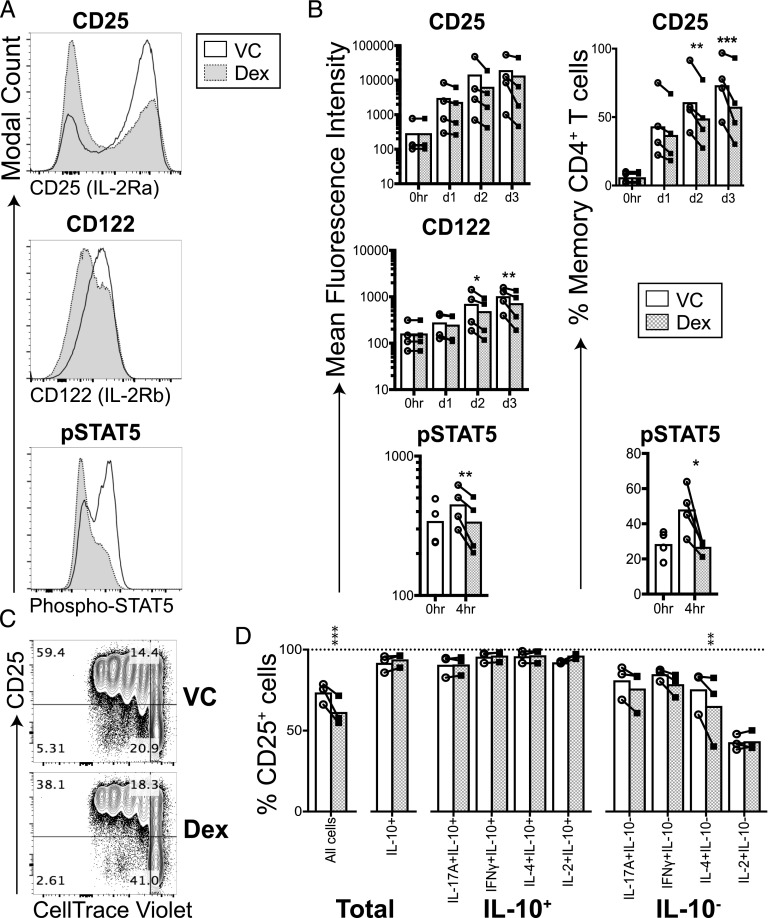
Glucocorticoids dampen IL-2 signaling in memory CD4^+^ T cells. Memory CD4^+^ T cells were stimulated in the presence of vehicle control (white) or 1 × 10^−7^M dexamethasone (Dex; gray). (**A** and **B**) After the indicated length of time, cells were surface stained for CD25 (IL-2Ra; top) and CD122 (IL-2Rb; middle); for phospho-STAT5 staining, cells were rested for 2 h, stimulated with 5 ng/ml rhIL-2 for 30 min, and then phospho-STAT5 expression was assessed. Shown are representative overlay histograms (A) alongside cumulative data for the mean fluorescence intensity and the percentage of positive cells [(B); *n* = 4]. (**C**) Representative plots showing expression of CD25 by cells relative to their proliferation status (CellTrace Violet) on day 5. (**D**) On day 5, cells were stimulated for 4 h with PMA and ionomycin prior to surface CD25 and intracellular cytokine staining (*n* = 3); shown is the percentage of CD25^+^ cells when gating on all cells (left) or the indicated cytokine-expressing populations (*n* = 3). Data assessed by a two-way ANOVA with Sidak multiple comparisons test. *p ≤ 0.05, **p ≤ 0.01, ***p ≤ 0.001.

IL-2 plays a central role in supporting the proliferation of T cells (30, 31), and its production is known to be inhibited by dexamethasone (32). Therefore, the contribution of this pathway to the observed differential effect of dexamethasone on IL-10^+^ versus IL-10^−^ cells was investigated next. Cell surface expression of IL-2R components CD25 (high-affinity IL-2Rα) and CD122 (IL-2Rβ) increased upon stimulation over time, and this increase was inhibited by dexamethasone (Fig. 5A, 5B). Dexamethasone-treated memory CD4^+^ T cells also showed lower amounts of downstream STAT5 phosphorylation as compared with vehicle control. The cells that had upregulated CD25 proceeded into cell division regardless of the action of dexamethasone (Fig. 5C). Moreover, the percentage of CD25^+^ cells was higher in the IL-10^+^ cells as compared with IL-10^−^ cells, suggesting that IL-10^+^ cells are selectively resistant to modulation of CD25 by dexamethasone (Fig. 5D). This provides a possible explanation for our findings that IL-10^+^ memory CD4^+^ T cells continue to proliferate in the presence of dexamethasone.

### High-dose IL-2 maintains dexamethasone-induced IL-10 while inhibiting IL-17A in memory CD4^+^ T cells

**FIGURE 6.**
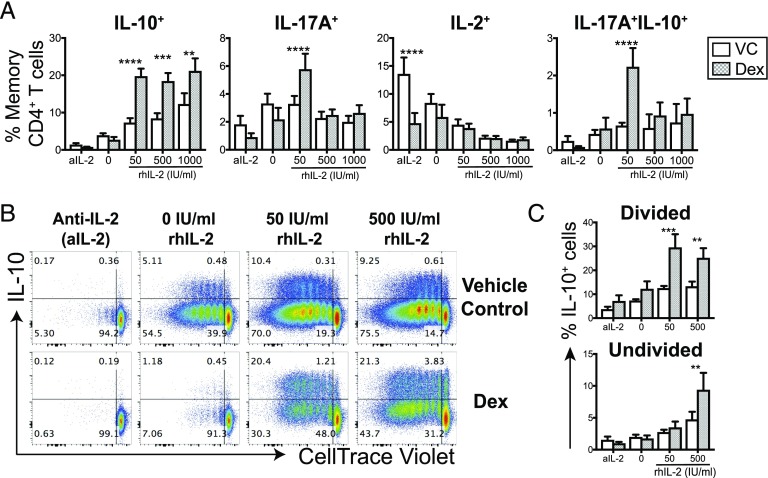
High-dose IL-2 in the context of glucocorticoid induces maximal IL-10 production while inhibiting IL-17A in memory CD4^+^ T cells. Memory CD4^+^ T cells were stimulated with anti-CD3 alone (0 IU/ml rhIL-2), plus 10 mg/ml neutralizing anti-IL-2 (aIL-2) or in the presence of the indicated concentration of rhIL-2 (50, 500, or 1000 IU/ml) in the presence of vehicle control (white bars) or 1 × 10^−7^M dexamethasone (Dex; gray bars). On day 5, cells were stimulated for 4 h with PMA and ionomycin prior to performing intracellular cytokine staining. (**A**) The percentage of cells expressing the indicated cytokine(s). (**B**) Representative plots showing expression of IL-10 relative to cell proliferation over the IL-2 titration. (**C**) The percentage of IL-10^+^ cells when gating on the divided (top) or undivided (bottom) cells (*n* = 6). **p ≤ 0.01, ***p ≤ 0.001, ****p ≤ 0.0001 between control and dexamethasone.

In the experiments described above, 50 IU/ml rhIL-2 was routinely added to all cultures. To further investigate the resistance of IL-10^+^ cells to downregulation of the IL-2R CD25 and continued proliferation in the presence of dexamethasone, we investigated the contribution of IL-2 to this process. Dexamethasone induction of IL-10 required the addition of rhIL-2, with no dexamethasone-mediated enhancement of IL-10 observed in the presence of anti–IL-2–neutralizing mAb because of its suppressive effect on proliferation (Fig. 6A, 6B). In the absence of dexamethasone, addition of anti–IL-2 mAb to the cultures led to a consistent decrease in the frequency of cells producing IL-10 and all effector cytokines measured, with the exception of IL-2, which was shown to be increased as compared with the vehicle control without IL-2 intervention (Fig. 6A; data not shown for IFN-γ and IL-4). Conversely, increasing concentrations of rhIL-2 in the absence of dexamethasone dose dependently increased the frequency of IL-10–producing cells while reducing that of IL-2–producing cells. Nevertheless, at all concentrations of IL-2 studied, 50–1000 IU/ml, the addition of dexamethasone further enhanced the frequency of memory CD4^+^ T cells producing IL-10 without affecting levels of IL-2 (Fig. 6A, 6B).

Enhancement of the frequency of IL-17A^+^ T cells by dexamethasone was also IL-2 dependent, as it was not observed either in the presence of anti–IL-2 or without addition of exogenous IL-2 to the cultures (Fig. 6A). In contrast to IL-10, however, the capacity of dexamethasone to increase IL-17A production was only seen at the lower concentration of IL-2 studied and was inhibited at the higher concentrations of IL-2 (500–1000 U/ml). Therefore, higher-dose rhIL-2 importantly maintained the elevated IL-10 response to dexamethasone while constraining the inductive effect of dexamethasone on IL-17A. As observed for IL-17A, low-dose, but not higher concentrations of rhIL-2 were required for dexamethasone to enhance the frequency of memory CD4^+^ T cells coproducing IL-10 and IL-17A (Fig. 6A). Furthermore, when higher doses of rhIL-2 was added to the cultures, dexamethasone increased IL-10 production by both dividing and undividing cells (Fig. 6B, 6C). Thus, our data suggest that IL-2 can independently upregulate IL-10 in the absence of dexamethasone, and that dexamethasone further increases IL-10 over and above the effect of IL-2. Notably, the combination of glucocorticoids and a higher dose of IL-2 promoted an IL-10^+^ memory CD4^+^ T cell population with minimal coproduction of proinflammatory cytokines.

## Discussion

Glucocorticoids are the cornerstone of treatment for patients with asthma and many other inflammatory conditions, and their clinical efficacy is linked, in part, to their capacity to induce synthesis of the anti-inflammatory cytokine IL-10 (9). To further expand our understanding of glucocorticoid action, we used cells from healthy individuals who respond to dexamethasone by increases in IL-10 and IL-17A but are devoid of any confounding asthma-associated defects (7). We show that a major source of glucocorticoid-induced IL-10 synthesis is CD4^+^ memory T cells, which are abundant in tissues such as the lung (33). Glucocorticoids enhanced the frequency of IL-10–expressing T cells, an effect that was particularly marked in T cells coexpressing IL-10 along with a range of effector, including Th17-associated, cytokines. The glucocorticoid dexamethasone concurrently increased the frequency of cells producing IL-17A, resulting largely from enrichment in the frequency of IL-17A^+^IL-10^+^ cells. Notably, cells producing IL-10 and/or IL-17A, including IFN-γ^+^IL-10^+^, but not IFN-γ^+^IL-10^−^, cells, were refractory to the antiproliferative effects of dexamethasone. This glucocorticoid-induced IL-10 production was dependent on IL-2, which, itself, could independently upregulate IL-10. Higher doses of rIL-2, while sustaining IL-10 production, inhibited the dexamethasone-induced IL-17A^+^ response.

IL-10–producing Th17 cells are indicative of the nonpathogenic Th17 cell population that has been shown to have a critical role in restraining Th17 cell–mediated inflammatory and autoimmune diseases (25–27). This contrasts with proinflammatory IFN-γ^+^Th17 cells that have been implicated in both murine (21, 22) and human (23) pathologies. In addition to inducing IL-10^+^IL-17^+^ T cells, we show in this study that dexamethasone also induced IL-10^+^IL-22^+^ cells. Because Th17-associated cytokines play a central role in defense against extracellular infections at mucosal sites, we propose that, in tissues such as the lung, the dual capacity of glucocorticoids to both maintain at least certain antimicrobial defense mechanisms via IL-17A and IL-22, as well as dampen inflammation via IL-10, is highly desirable. It would, therefore, be interesting to study the coproduction of these cytokines over the course of a range of diseases in humans; in mice, IL-22 has been shown to drive the initiation of airway inflammation but act as a negative regulator in established disease (34). We also observed that dexamethasone selectively enriched a population of IFN-γ^+^IL-10^+^ cells, which we hypothesize would dampen excessive airway inflammation. Indeed, our earlier studies in steroid-refractory patients indicate that both IL-17A and IFN-γ, but not IL-10, synthesis are significantly elevated compared with steroid-sensitive patients (12).

The capacity of dexamethasone to enhance the frequency of cells coproducing IL-10 with Th17-associated cytokines reflected preferential outgrowth of this population in culture and was dependent on the presence of the growth factor IL-2. Only the lower concentration of IL-2 studied promoted the expansion of IL-17A^+^ or IL-17A^+^IL-10^+^ cells. Low-dose IL-2 is required for T cell proliferation and survival (30, 31), but increased IL-2 signaling is proposed to impair IL-17A responses as p-STAT5 is reported to compete with p-STAT3 for binding to the *Il17a* gene locus (35, 36). Therefore, the fact that dexamethasone downregulated, but importantly did not eliminate, expression of IL-2, IL-2Rα1, IL-2Rβ, and p-STAT5 may favor the outgrowth of Th17 cells. However, we show that higher doses of IL-2 overcame this to inhibit dexamethasone-induced IL-17A although sustaining IL-10–producing cells. Hence, our findings provide a novel approach to promoting nonpathogenic Th17 cells, which coproduce IL-10. Of note, as well as driving the outgrowth of IL-10^+^ cells, the higher concentrations of studied rhIL-2 promoted the expansion of a population of cells that were predominantly negative for all cytokines assessed (data not shown) and may reflect a central memory T cell population (37).

The mechanisms by which dexamethasone increases the synthesis of IL-10 and IL-17A are likely to be distinct given the pleiotropic nature of glucocorticoid actions. The relatively slow nature of both responses suggests that they are unlikely to be due to a dexamethasone/GR complex binding directly to target DNA, although glucocorticoid response elements [consensus motif from Hocomoco, Jaspar (38)] are located both up- and downstream of the *IL10* promoter. Glucocorticoid response elements are also found within the vicinity of the *IL2*, *IL2RA*, *IL2RB*, and *STAT5* loci, suggesting that dexamethasone may directly influence the transcription of these genes. Increased production of Th17-associated cytokines may also be, in part, due to reduced Th1 and Th2 responses, both of which have been shown to reduce IL-17A production (39, 40). Using the same experimental system, we previously reported that addition of IL-4 to the culture attenuated the IL-17A response (12). Conversely, neutralizing IL-4, but not IFN-γ, in culture increased levels of IL-17A in the control condition to levels comparable of dexamethasone-treated cells, which was unaffected by IL-4 blockade (data not shown).

Minimal suppression by glucocorticoids of Th17 cell proliferation, as shown in this study, has also been observed in murine in vitro and in vivo systems when compared with Th1 and Th2 cells (18, 41), and human proinflammatory Th17 cells have been shown to be refractory to dexamethasone’s suppressive effects on proliferation (23). One potential mechanism for the altered proliferative response identified in this study is that cells coproducing IL-17A and IL-10 expressed elevated levels of CD25, which were refractory to inhibition by dexamethasone, in contrast to cells not making IL-10, enabling their preferential expansion. In line with this observation, it has been shown that only a subset of CD4^+^ T cells expressing elevated levels of CD25 continue to proliferate in the presence of dexamethasone, although cytokine expression was not assessed in that study (42). Our novel findings show that IL-10^+^ cells, irrespective of whether they coproduce proinflammatory cytokines, can maintain IL-2 signaling and proliferate in the presence of glucocorticoids.

Our previous studies identify glucocorticoid-induced IL-17A as a feature of SR severe asthma (12), but in this study, we address the underlying mechanisms and single-cell phenotyping. Our data suggest that aberrant glucocorticoid-driven IL-17A in SR asthma may be a result of the immune environment in these patients, and, in particular, low, but not absent, levels of IL-2. Indeed, daily glucocorticoid therapy might dampen IL-2R expression and signaling to favor Th17 differentiation and/or expansion, particularly in the setting of weak T cell stimulation, such as during subclinical airways infection with microbes such as *Haemophilus*, which is associated with severe asthma (43). To this end, severe asthmatics were found to have elevated soluble CD25 in circulation and increased expression by CD4^+^ T cells, which inversely correlated with airway obstruction (44, 45).

Although not studied in asthma, low-dose IL-2 therapy has been found beneficial in patients with diseases including graft-versus-host disease, many of whom were receiving glucocorticoids (46), and hepatitis C virus–induced vasculitis (47). Such beneficial effects may be due to IL-2 dampening Th17 cells, which is hypothesized to be beneficial in graft-versus-host disease (48). However, much of the research emphasis has been in describing the expansion of Foxp3^+^ Tregs following IL-2 therapy (46, 49, 50). Coexpression of Foxp3 and IL-10 was not assessed in this study, but dexamethasone-driven IL-10 was not accompanied by a change in Foxp3 mRNA or protein expression (data not shown), suggesting that the cells in this study were not conventional Tregs. This is in line with both our own and independent reports (51–53) that observe very low levels of coexpression of Foxp3 and IL-10 protein in human CD4^+^ T cells. Our findings, showing that IL-2 increases IL-10–producing memory CD4^+^ T cells, may also provide an explanation for the defective IL-10 production, but normal Foxp3 gene expression, in CD4^+^ T cells from a patient with CD25 deficiency, manifesting as immune dysregulation, polyendocrinopathy, enteropathy, and X-linked–like syndrome (54).

We have previously shown that vitamin D can restore dexamethasone-induced IL-10 production in severe asthma (10, 12), but whether combining IL-2 therapy with glucocorticoids to further enhance IL-10 production and suppress IL-17A production is a pharmacotherapeutic option in severe asthma needs further consideration. First, IL-17A, and other Th17-associated cytokines, have both beneficial and harmful actions. Second, one of the main limitations to IL-2 therapies is the profound differences in immunologic effects of different IL-2 doses, with proinflammatory actions observed at the highest doses. Interestingly, Sockolosky and colleagues (55) recently published on the generation of orthogonal IL-2 cytokine receptor complexes that selectively target cell populations for adoptive cell therapy. Although the study did not measure its effects on levels of IL-10 and Th17-associated cytokines, they showed that IL-2, but not the orthogonal IL-2 complex, increased the serum levels of several proinflammatory cytokines in mice, which is associated with serious side effects. It would, therefore, be interesting to investigate whether this may be a mechanism to therapeutically skew responses toward a single IL-10–producing response, and any impact of common therapeutic agents, such as glucocorticoids, upon this process.

In summary, we show that dexamethasone promotes the preferential proliferation of IL-10^+^ and IL-17A^+^ memory CD4^+^ T cells, specifically enriching for populations of cells coproducing IL-10 and multiple proinflammatory cytokines. Increasing the concentration of rhIL-2 in the presence of dexamethasone maintained elevated levels of IL-10 while reducing the IL-17A response, which may provide new avenues for the development of improved clinical therapies for asthma.

## Supplementary Material

Data Supplement
