# Supplementary material for: High-Dose IL-2 Skews a Glucocorticoid-Driven IL-17+IL-10+ Memory CD4+ T Cell Response towards a Single IL-10–Producing Phenotype
Source: J Immunol. 2018 Dec 31;202(3):684–93. doi: 10.4049/jimmunol.1800697 (PMC6341182; doi:10.4049/jimmunol.1800697)
Supplement: Data Supplement [file JI_1800697.zip › JI_1800697_Supplemental_Figures_1.pdf]

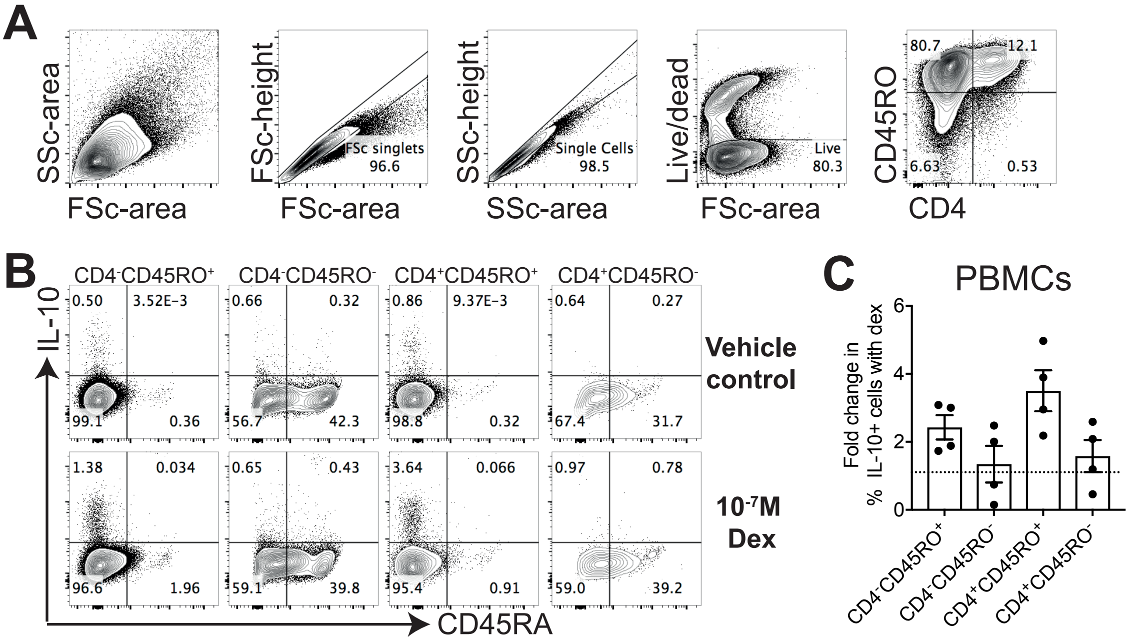

Supplementary Figure 1: Glucocorticoids enrich the proportion of IL-10+ within the memory CD4+ T cells compartment.
PBMCs were stimulated with 10-7M dexamethasone or vehicle control for 5 days. Cells were then stimulated for 4 hours with PMA and Ionomycin prior to staining for surface markers and intracellular IL-10. The percentage of the indicated sub-population of cells expressing IL-10 was determined based on expression of CD4 and CD45RO (A - gating strategy). Shown are representative plots (B) and cumulative data showing the fold change in percentage IL-10+ cells with dexamethasone treatment is normalised to the vehicle control (C; n=4).

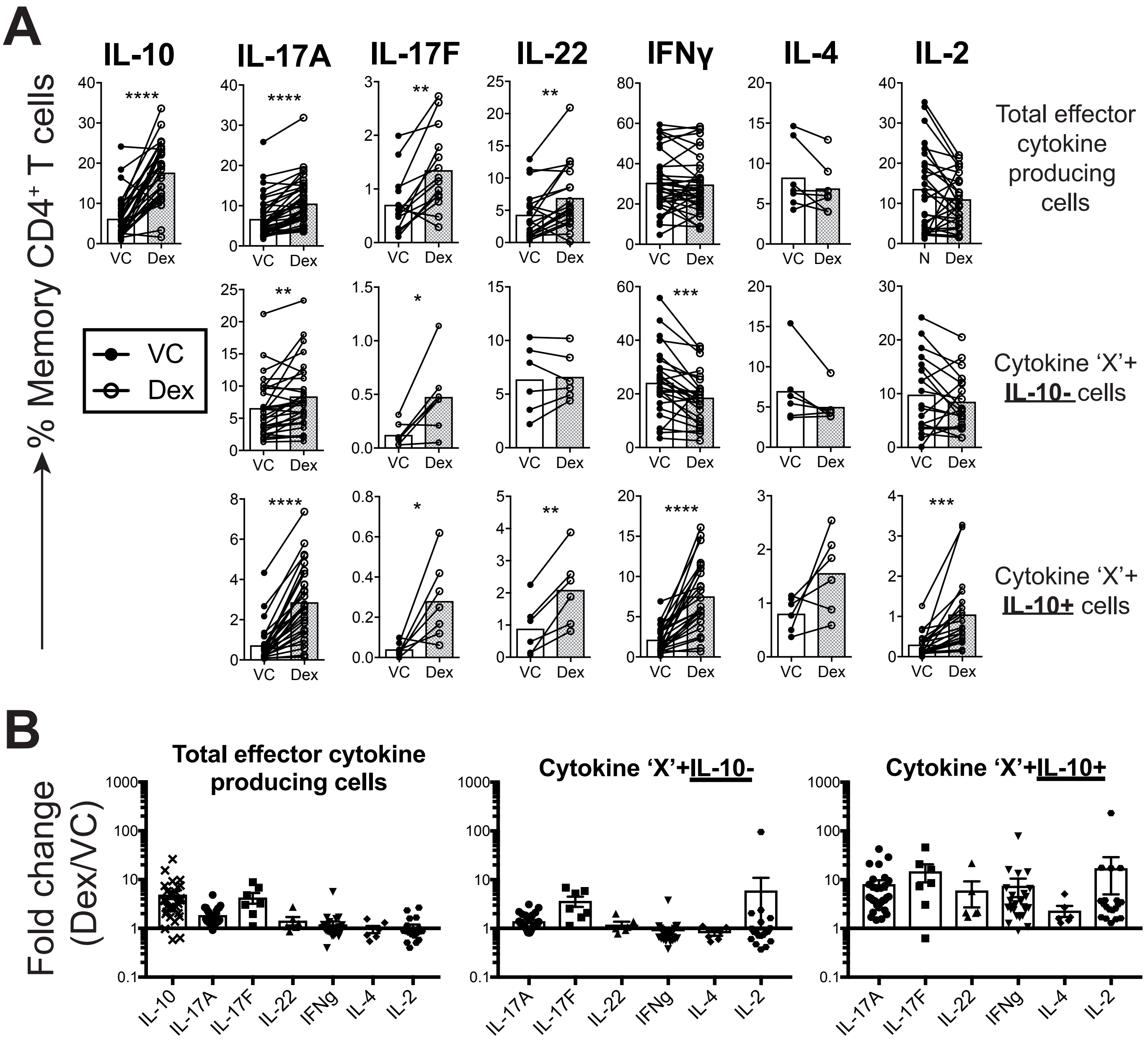

Supplementary Figure 2: Glucocorticoid specifically enrich the proportion of cells co-producing IL-10 and multiple effector cytokines.

Memory CD4+ T cells were stimulated in the presence of vehicle control or 10-7M dexamethasone. On day 5 cells were stimulated for 4 hours with PMA and Ionomycin prior to performing intracellular cytokine staining. A, the percentage of memory CD4+ T cells producing the indicated cytokine, presented as total (top), IL-10- (middle) and IL-10+ (bottom) for each donor (IL-10 n=32; IL-17A n=29; IL-17F n=7; IL-22 n=6; IFN n=25; IL-4 n=6; IL-2 n=19). Data assessed by paired-t-test; * p ≤ 0.05, ** p ≤ 0.01, *** p ≤ 0.001, **** p ≤ 0.0001. B, fold change in the frequency of cytokine producing cells in dexamethasone-treated cultures as compared to the vehicle control for total (left), IL-10- (middle) and IL-10+ (right) cells.
